# P-1320. HAISeq: Monitoring Emerging and Novel Gram-Negative Antimicrobial Threats from Healthcare Settings in the United States with Publicly Available NCBI BioProject Data

**DOI:** 10.1093/ofid/ofae631.1499

**Published:** 2025-01-29

**Authors:** Kara A Moser, Alyssa Kent, Jill V Hagey, Gillian A McAllister, Rachael Aubert, Alison L Halpin

**Affiliations:** Division of Healthcare Quality Promotion, Centers for Disease Control and Prevention; CDC, Atlanta, Georgia; Clinical and Environmental Microbiology Branch, Division of Healthcare Quality Promotion, Centers for Disease Control and Prevention, Atlanta, Georgia; Centers for Disease Control and Prevention, Atlanta, GA; AR Coordination and Strategy Unit, Division of Healthcare Quality Promotion, Centers for Disease Control and Prevention, Atlanta, Georgia; Centers for Disease Control and Prevention, Atlanta, GA

## Abstract

**Background:**

Gram-negative healthcare-associated infections (HAIs) and antimicrobial resistance (AR) cause considerable morbidity and mortality in the United States. Whole genome sequencing (WGS) has become a critical methodology to describe HAIs, providing important contextual information on resistance genes and relatedness. It is essential that public health agencies leverage large amounts of WGS data quickly for public health action, and that data can be accessible across jurisdictions.

**Methods:**

In advance of an influx of resources to state public health laboratories for HAI/AR WGS capacity, CDC’s Division of Healthcare Quality Promotion (DHQP) established the HAISeq Umbrella BioProject in the National Center for Biotechnology Information’s Short Read Archive as a centralized repository for WGS data generated by DHQP and partners. Data submitted is from CDC’s Antimicrobial Resistance Laboratory Network, surveillance projects, and outbreak investigations.

**Results:**

The gram-negative-specific BioProject within the HAISeq Umbrella currently contains 6.5TB of WGS data, representing > 20,000 gram-negative isolates, ∼40 genera, and > 450 unique AR genes. While DHQP priorities are heavily reflected in represented genera (*Acinetobacter*, *Pseudomonas*, *Klebsiella, Escherichia*) and gene targets (e.g., >10 carbapenemase families), the dataset also represents hundreds of minor frequency species and genes captured from the above sequencing efforts. DHQP uses bimonthly data pulls to detect potential novel and emerging HAI/AR genetic threats. Examples include identification of novel carbapenemase variants (e.g., 23 variants from all “Big 5” groups), variants that convey unexpected phenotypes to treatment options (e.g., carbapenem susceptible but ceftazidime-avibactam resistant), and rare gene/organism combinations (e.g., *_bla_*OXA-48-like genes in *Acinetobacter baumannii*). Data are also used to rapidly provide feedback to public health laboratories on data quality issues. Finally, these data help inform national HAI/AR sequencing priorities.

**Conclusion:**

This publicly available dataset, alone and in combination with traditional testing data, allows for both federal and state public health labs to leverage WGS for public health action while maintaining data privacy.

**Disclosures:**

**All Authors**: No reported disclosures

